# Early life swimming pool exposure and asthma onset in children – a case-control study

**DOI:** 10.1186/s12940-018-0383-0

**Published:** 2018-04-11

**Authors:** Martin Andersson, Helena Backman, Gunnar Nordberg, Annika Hagenbjörk, Linnea Hedman, Kåre Eriksson, Bertil Forsberg, Eva Rönmark

**Affiliations:** 1 Department of Public Health and Clinical Medicine, Occupational and Environmental Medicine, Umeå University, S-90187 Umeå, Sweden; 2 The OLIN studies, S-97189 Luleå, Sweden

**Keywords:** Asthma, Trichloramine, Children, Swimming

## Abstract

**Background:**

Trichloramine exposure in indoor swimming pools has been suggested to cause asthma in children. We aimed to investigate the risk of asthma onset among children in relation to individual trichloramine exposure.

**Methods:**

A longitudinal nested case-control study of 337 children with asthma (cases) and 633 controls aged 16–17 years was performed within a population-based cohort from The Obstructive Lung Disease in Northern Sweden studies (OLIN). Year of asthma onset and exposure time at different ages were obtained in telephone interviews. Trichloramine concentrations in the pool buildings were measured. Skin prick test results for inhalant allergens were available from previous examinations of the cohort. The risk for asthma was analyzed in relation to the cumulative trichloramine exposure before onset of asthma.

**Results:**

The participation rate was high in the original cohort (88 to 96%), and in the case-control study (80%). Trichloramine concentrations ranged from 0.020 to 0.55 mg/m^3^ (mean 0.15 mg/m^3^). Swimming pool exposure in early life was associated with a significantly higher risk of pre-school asthma onset. A dose-response relationship between swimming pool exposure and asthma was indicated in children with asthma onset at 1 year of age. Children who were both sensitized and exposed had a particularly high risk.

**Conclusions:**

Early life exposure to chlorinated swimming pool environments was associated with pre-school asthma onset.

**Electronic supplementary material:**

The online version of this article (10.1186/s12940-018-0383-0) contains supplementary material, which is available to authorized users.

## Background

There are several preventable environmental factors known to increase the risk of asthma in children, e.g. exposure to vehicle exhaust [1, 2], damp and moldy housing [3], and tobacco smoke [4, 5]. Trichloramine is formed in chlorinated swimming pool water following a reaction between sodium hypochlorite and nitrogen containing substances [6]. Trichloramine is an eye and airway irritant [7], but is also considered to be a possible cause of asthma in children [7–13]. It has been suggested that children may be most vulnerable to trichloramine exposure early in life, and that sensitization to inhalant allergens may interact with swimming pool exposure in causing asthma [8, 10–14]. Sensitization is related to persistence of childhood asthma into adolescence and adulthood [15, 16], making prevention of this asthma subgroup especially important. Because swimming in general has positive effects on children’s health through physical activity, avoiding swimming should not be recommended without clear scientific evidence.

The findings on the association between asthma and swimming pool exposure are not conclusive, and there is a need for population-based studies using individual exposure assessment to clarify the potential risks [7]. Although objective exposure measurements are considered essential [7], this has seldom been performed, especially in studies reporting no increased risk [14, 17–19]. In a previous cross-sectional study within the Obstructive Lung Disease in Northern Sweden studies (OLIN), we found an association between asthma and swimming pool attendance among sensitized children [13]. In the present case-control study, we used an epidemiological design allowing estimation of exposure before asthma onset and included measurements of trichloramine in swimming pools facilities. We aimed to evaluate the risk of asthma onset in children in relation to trichloramine exposure before onset of asthma.

## Methods

### Study design

**Fig. 1:**
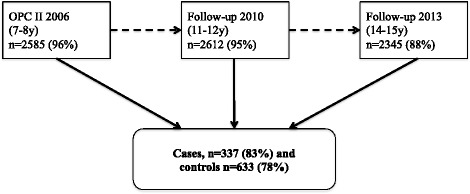
Flow-chart of the second OLIN pediatric cohort. Cases were children with physician-diagnosed asthma at either the original survey at age 7–8 years or at the follow-ups at 11–12 and 14–15 years. Controls consisted of children without asthma, allergic rhinitis and asthma symptoms in any of the surveys

We conducted a nested case-control study of 337 children with asthma and 633 controls aged 16–17 years within the population-based second OLIN pediatric cohort [20], briefly described below. The cases were selected based on the reporting of physician-diagnosed asthma at any of the three surveys within the cohort from 2006 to 2013 at ages 7–8, 11–12 and 14–15 years. The double number of controls compared to cases, were randomly selected among children without asthma, allergic rhinitis and asthma symptoms in any of the cohort surveys (Fig. 1). Cases and controls were not individually matched.

The parents of cases and controls were telephone interviewed together with their children by a specifically trained registered nurse during 2014 and 2015. She recorded indoor swimming pool exposure time at different ages (year by year from birth), airway and eye symptoms, and in children with physician-diagnosed asthma also age at first onset of asthma symptoms. The participation rates were high both in the original cohort (96%), the follow-up of the cohort (95% in 2010 and 88% in 2013), and in the present case-control study (80%). Only a few of invited but non-responding children declined taking part in the study, the majority were not possible to reach by phone (Table 1). An occupational health and safety engineer measured trichloramine concentrations from January to April 2015. We investigated if trichloramine exposure until a certain age was higher for children with asthma symptom onset after that age (either in the directly following year or during several years), using cumulative exposure for all controls until the same age as reference.

**Table 1:** Prevalence (%) of demographics and conditions among responders and non-responders in the current case-control study

Cases, *n* = 405	Responders (*n* = 337)	Non-responders (*n* = 68)	p
Sex (boy)	54.6%	54.4%	0.98
Current wheeze	47.6%	42.4%	0.44
Any SPT+ at age 12y	64.3%	58.7%	0.48
Paternal smoking	16.0%	7.8%	0.09
Maternal smoking	13.5%	10.6%	0.53
Own smoking^a^	5.7%	7.5%	0.59
Controls, *n* = 809	Responders (*n* = 633)	Non-responders (*n* = 176)	p
Sex (boy)	47.9%	54.0%	0.15
Current wheeze	0.3% (*n* = 2)	1.2% (n = 2)	0.17
Any SPT+ at age 12y	20.1%	18.7%	0.77
Paternal smoking	10.8%	18.0%	0.01
Maternal smoking	12.8%	14.9%	0.49
Own smoking^a^	5.1%	5.7%	0.75

Based on data reported in the questionnaire survey of the entire cohort at 14–15 years of age and the skin prick tests performed at age 11–12 years. The proportion of missing values on the questionnaire questions was < 4%

^a^More than “never” or “almost never”

### The second OLIN pediatric cohort

The second OLIN pediatric cohort was recruited in 2006. All children in the first and second grade of primary school (aged 7–8 years) in the municipalities Luleå, Kiruna and Piteå were invited, and 2585 (96%) participated [20, 21]. The parental questionnaire [20] included ISAAC (The International Study on Asthma and Allergy in Childhood) questions on asthma and allergy symptoms [22] with added questions on environment, heredity and physician-diagnosis of asthma [20]. Self-reporting of a physician-diagnosis of asthma among 7–8 year old children in this study area has been validated by a structured pediatric assessment. The sensitivity was 70% and the specificity > 99% [23]. In the follow-up in 2010 (the children now at age 11–12 years), parents answered the same questionnaire as in 2006. The cohort was also followed-up in 2013 (at age 14–15 years) using the same methodology.

### Skin prick testing

Skin prick testing (SPT) followed the European Academy of Allergy and Clinical Immunology (EAACI) method and was performed in two of the municipalities (Luleå and Kiruna), both in 2006 (at 7–8 years of age, *n* = 1700, 90% of invited), and in 2010 (at 11–12 years, *n* = 1652, 89% of invited) [24]. Ten inhalant allergens were evaluated (birch, timothy, mugwort, cat, dog, horse, two molds, and two mites). A mean wheal diameter ≥ 3 mm was defined as positive. SPT results were available for 64% of cases and 67% of controls. The agreement between SPT and serum-IgE testing was high in this cohort [20].

### Trichloramine exposure

#### Sampling and analysis

Stationary air sampling of trichloramine (NCl_3_) was performed at two different occasions approximately 2–3 days apart, at the 7 most frequently visited indoor swimming pools, from January to April 2015. In two swimming pools air samples collected at earlier occasions were used. No measurements were available at four premises. Air sampling was performed during winter or spring as the swimming pools are closed during the summer, and at day time as the children mostly attended the swimming pools during the day. Sodium hypochlorite has been used as the disinfection agent in all of the premises during the study time period. The pH in the swimming pool water was 7.3–7.5 with a water temperature between 28 and 37 °C. The concentration of free and combined chlorine in the pool water was 0.4–1.1 mg Cl_2_/L and 0.07–0.6 mg Cl_2_/L, respectively, which is within the Swedish limits. The total number of swimmers present in the pool during the 4 h sampling period was 4–168. There have been no major changes in the ventilation system during the study time period (personal communication with the technicians at the swimming pools).

The sampling equipment was placed at one to four different spots within the pool area, 0.3 m above the floor level and 0.5 m from the water surface. The sampling time was in general 180 min, with a change of filters after 90 min for evaluating possible variation in trichloramine concentration during the sampling session. Analysis of trichloramine was done according to Héry et al. [6, 25]. In brief, air passed through a sampling filter by means of a pump. The filter was impregnated with sodium carbonate and diarsenic trioxide. Trichloramine is reduced to chloride ions (Cl^−^) on the impregnated filters. After sampling, the filters were desorbed in water, sonicated and filtered, and the chloride ions were analysed in a suppressed ion chromatography system. Control samples of known chloride concentrations and at least two blank samples were run together with the samples in each run. The analyses were performed at Occupational and Environmental Medicine, Umeå University. (Detailed information of sampling and analysis can be found in Additional file 1).

#### Exposure assessment

Trichloramine concentration in the air of swimming pool buildings, in combination with interview-based information about the frequency of attendance at different ages and which pool(s) the children visited, was used to estimate cumulative exposure to NCl3 for each participating child. Cumulative exposure was calculated year by year (from birth) as the number of hours spent in indoor swimming pools multiplied by the mean trichloramine concentration at the respective pool. Therefore, cumulative exposure before asthma onset, or before the corresponding age for controls, was obtained. The cumulative exposure was either divided into unexposed or exposed or into three groups; unexposed, the children within the lowest two tertiles of cumulative exposure and the children within the highest tertile of cumulative exposure.

### Definitions

*Allergic sensitization (SPT+):* Any positive SPT at age 7–8 years or 11–12 years.

*Maternal smoking:* Mother smoking during pregnancy or at any time until the child was 10 years old [20].

*Family history of asthma:* Parents or siblings with asthma [20].

*Pre-school onset asthma:* Cases with onset of first asthma symptom from 0 to 6 years of age.

*School-age onset asthma:* Cases with onset of first asthma symptom from 7 to 15 years of age.

*Cumulative exposure:* Cumulative number of hours spent in indoor swimming pools multiplied by mean trichloramine air concentration, calculated year by year from birth. In the analyses, only cumulative exposure before the asthma onset was taken into account, and the corresponding period was used for controls.

### Statistics

In bivariate comparisons of proportions, the Chi-square test was used. A *p*-value < 0.05 was considered statistically significant. Multinomial logistic regression models were applied to analyze the association between exposure in the first year of life and asthma onset between 1 and 2 years. Correspondingly, exposure in the first two years of life and asthma onset between 2 and 3 years was analysed, as well as exposure in the first three years of life and asthma onset between 3 and 4 years. In another model, the relation between pre-school and school-age onset asthma, respectively, and cumulative swimming pool exposure at different ages was analyzed, only including cases with a later asthma symptom onset than that age. As an example, when analyzing the association with cumulative exposure until the age of 1 year, cases with asthma symptom onset before their first birthday were excluded, and when analyzing the association with cumulative exposure until the age of 2 years, cases with asthma symptom onset before their second birthday were excluded. The regression models were used to estimate odds ratios (OR) with 95% confidence intervals (CI) for different exposure variables: a) exposed versus unexposed as reference, b) divided into three categories: high exposure (highest tertile of exposure), low-to-intermediate exposure (lowest two tertiles of exposure), and unexposed as reference, and c) divided into four categories based on exposure and allergic sensitization: exposed with SPT+, unexposed with SPT+, exposed with SPT-, and unexposed with SPT- (reference category). In the analyses including allergic sensitization (c), only children from the municipalities where SPT was performed could be included. The analyses were performed both unadjusted and adjusted for sex, family history of asthma, maternal smoking, and municipality. Spearman’s test was used for analysing correlations between free chlorine, bound chlorine and the number of children attending the swimming pool.

### Sensitivity analyses

Sensitivity analyses were performed regarding the association between swimming pool exposure and asthma onset: 1) with cumulative exposure calculated as hours multiplied by maximum concentrations of trichloramine (instead of mean-concentrations), 2) with cumulative exposure based on hours only without taking the objective measurements into consideration, 3) without adjusting the regression models for municipality, 4) with cumulative exposure based only on swimming pools where measurements of trichloramine were available and 5) with exposure based only on objective measurements of trichloramine without taking the number of hours into account.

## Results

The participants and the non-participants in the case-control study had very similar demographics and differed only regarding the prevalence of paternal smoking and family history of asthma and allergy (Table 1). The prevalence of potential risk factors among cases and controls at age 7-8y are presented in Additional file 2. Table 2 presents the number of children with asthma onset in pre-school age year by year (50% of all cases had pre-school asthma onset).

**Table 2:** Age at asthma symptom onset among cases

Age at asthma symptom onset		Number	Percent
Pre-school onset	< 1 years of age	15	4.5%
	1 year	60	17.8%
	2 years	21	6.2%
	3 years	17	5.0%
	4 years	16	4.7%
	5 years	13	3.9%
	6 years	26	7.7%
School-age onset	7–15 years	169	50.1%
All cases		337	100.0%

The trichloramine concentrations in the included swimming pools showed clear differences and ranged from 0.020 to 0.55 mg/m^3^ (arithmetic mean for all swimming pools 0.15 mg/m^3^) and are presented in Table 3. The concentration of free chlorine (Spearman’s r 0.62, *p* < 0.001) and the concentration of bound chlorine (Spearman’s r 0.30, *p* < 0.05) were correlated to the air concentration of trichloramine. The air concentration of trichloramine was not significantly affected by the number of swimmers present during sampling (Spearman’s r 0.24, *p* = 0.079) or the differing water temperatures (Spearman’s r 0.11, *p* = 0.424).

**Table 3:** Air concentrations of trichloramine at the different swimming pool facilities

	Swimming pool		NCl_3_ (mg/m^3^)						Hours	Proportion
Municipality	identification number	N	Mean	SD	Median	Min-Max			N^a^	%^b^
Kiruna	1	11	0.290	0.151	0.260	0.100	–	0.550	52,426	18.2%
Luleå	1	8	0.269	0.095	0.275	0.160	–	0.370	49,781	17.2%
Luleå	2	8	0.051	0.021	0.045	0.030	–	0.090	41,979	14.5%
Luleå	3	8	0.143	0.027	0.145	0.100	–	0.170	30,305	10.5%
Luleå	4	8	0.149	0.029	0.145	0.120	–	0.190	10,092	3.5%
Piteå	1	8	0.066	0.038	0.050	0.020	–	0.110	35,866	12.4%
Piteå	2	8	0.058	0.013	0.055	0.040	–	0.080	22,403	7.8%
Piteå	3	8	0.089	0.011	0.090	0.070	–	0.100	15,772	5.5%
Piteå	4	10	0.197	0.052	0.189	0.133	–	0.281	2529	0.9%
Other^c^		77	0.152	0.110	0.120	0.020	–	0.550	27,658	9.6%

*N* Number of measurements at each swimming pool facility

^a^The total number of hours spent in indoor swimming pools until age 15 by children in the study

^b^Proportion of the total number of hours spent in indoor swimming pools until age 15 by children in the study

^c^Data for four “other” swimming pool facilities where no air concentration measurements were available are based on the 77 samples collected at the nine participating facilities with objective data

At the telephone interview, 1.5% of the children with asthma (cases) reported that they had stopped attending indoor swimming pools because of asthma. Cases also reported significantly more eye irritation in indoor swimming pools (21.7%) compared to controls (9.0%). Some children with asthma had worsened asthma symptoms in indoor swimming pools (5.5%), of which all were sensitized to at least one inhalant allergen.

A significant association between the cumulative exposure levels in early childhood and development of pre-school asthma was found (Table 4). Unadjusted analyses are presented in Additional file 3. The results were similar when using the cumulative number of hours only (Table 5), max instead of mean trichloramine level (Additional file 4), when cumulative exposure was based only on swimming pools with objective measurements of trichloramine (Additional file 5), and when not including municipality in the analysis (Additional file 6). Also when exposure was based only on level of concentrations of trichloramine, the results were similar (Additional file 7).

**Table 4:** Adjusted OR for pre-school asthma and school-age asthma, respectively, in relation to cumulative exposure before asthma onset (Unexposed as reference)

Pre-school asthma							School-age asthma		
	Low-to intermediate exposure		High exposure		Any Exposure		Any Exposure		
Age	OR	(95% CI)	OR	(95% CI)	OR	(95% CI)	Age	OR	(95% CI)
1y (*n* = 153)	1.97	(1.26–3.07)	1.93	(1.12–3.33)	1.95	(1.33–2.86)	1y (n = 169)	1.25	(0.87–1.80)
2y (*n* = 93)	2.07	(1.23–3.48)	1.42	(0.73–2.76)	1.82	(1.14–2.90)	2y (*n* = 169)	1.19	(0.83–1.69)
3y (*n* = 72)	1.93	(1.08–3.44)	1.26	(0.60–2.64)	1.68	(0.98–2.86)	3y (n = 169)	1.24	(0.87–1.77)
4y (*n* = 55)	1.25	(0.65–2.41)	1.07	(0.47–2.43)	1.19	(0.65–2.20)	4y (n = 169)	1.13	(0.78–1.65)
5y (*n* = 39)	1.19	(0.53–2.67)	1.01	(0.39–2.61)	1.13	(0.53–2.39)	5y (n = 169)	1.09	(0.72–1.64)
6y (*n* = 26)	0.81	(0.27–2.43)	1.21	(0.38–3.91)	0.94	(0.34–2.65)	6y (n = 169)	1.21	(0.73–2.00)

Analysis at 1 years = the relationship between exposure in the first year of life and asthma onset between 1 and 6 years of age (pre-school asthma), and between 7 and 15 years of age (school-age asthma), respectively. Analysis at 2 years = the relationship between exposure in the first two years of life and asthma onset between 2 and 6 years of age, and between 7 and 15 years of age, respectively. Analysis at 3 years = the relationship between exposure in the first three years of life and asthma onset between 3 and 6 years of age, and between 7 and 15 years of age, etc

**Table 5:** Adjusted OR for pre-school asthma vs controls in relation to the number of hours spent in swimming pools before asthma onset (Unexposed as reference)

	Low-to intermediate exposure			High exposure			Any exposure		
Age	OR	(95% CI)		OR	(95% CI)		OR	(95% CI)	
1y (n = 153)	2.25	(1.39	3.65)	1.73	(1.08	2.76)	1.95	(1.33	2.86)
2y (n = 93)	1.86	(1.10	3.16)	1.75	(0.94	3.25)	1.82	(1.14	2.90)
3y (n = 72)	1.82	(1.01	3.29)	1.48	(0.75	2.91)	1.68	(0.98	2.86)
4y (n = 55)	1.23	(0.63	2.40)	1.14	(0.53	2.42)	1.19	(0.65	2.20)
5y (n = 39)	0.89	(0.38	2.07)	1.51	(0.64	3.56)	1.13	(0.53	2.39)
6y (n = 26)	0.94	(0.31	2.78)	0.95	(0.29	3.09)	0.94	(0.34	2.65)

*Exposure = Cumulative number of hours*

Analysis at 1 years = the relationship between exposure in the first year of life and asthma onset between 1 and 6 years of age. Analysis at 2 years = the relationship between exposure in the first two years of life and asthma onset between 2 and 6 years of age. Analysis at 3 years = the relationship between exposure in the first three years of life and asthma onset between 3 and 6 years of age, etc

**Fig. 2:**
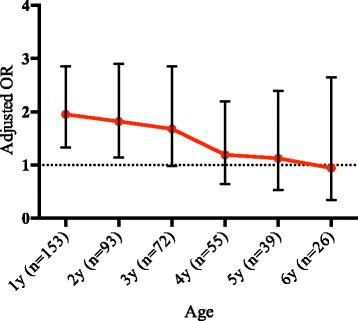
Odds ratios (OR) for pre-school asthma, in relation to exposure (exposed or unexposed) before asthma onset. The OR:s at the different ages are estimated by only including cases with asthma onset at or after that specific age

The association of exposure (exposed or unexposed) until ages 1–6 years, respectively, with later pre-school onset asthma (*n* = 168) is shown in Fig. 2. Exposure before 1 year of age was significantly associated with pre-school asthma with onset at age 1 or later (adjusted OR 1.95, 95%CI 1.33–2.86), and exposure before the age of 2 years was associated with pre-school asthma with onset at age 2 or later (Fig. 2). School-age onset asthma (*n* = 169) was not associated with pre-school swimming pool exposure (Table 4).

The risk of pre-school asthma onset among children who participated in SPT testing, in relation to exposure before the age of asthma onset and sensitization status is showed in Table 6. The risk of asthma onset was markedly increased by swimming pool exposure, but only among sensitized children.

**Table 6:** Adjusted OR for pre-school asthma in relation to exposure before asthma onset by allergic sensitization

	Exposed with SPT-				Unexposed with SPT+				Exposed with SPT+			
	OR	(95% CI)			OR	(95% CI)			OR	(95% CI)		
1y (*n* = 102)	1.69	(0.78	–	3.67)	8.22	(4.32	–	15.64)	13.68	(6.44	–	29.08)
2y (*n* = 62)	0.96	(0.30	–	3.08)	9.77	(4.00	–	23.90)	19.85	(7.77	–	50.71)
3y (*n* = 48)	1.29	(0.33	–	5.04)	11.47	(3.39	–	38.84)	24.14	(7.57	–	76.96)
4y (*n* = 35)	0.83	(0.13	–	5.20)	12.62	(2.43	–	65.44)	24.43	(5.29	–	112.80)
5y (n = 26)	0.37	(0.05	–	2.79)	10.16	(1.85	–	55.91)	11.38	(2.35	–	55.12)
6y (*n* = 18)	0.61	(0.06	–	6.25)	6.66	(0.52	–	84.91)	9.10	(1.09	–	76.35)

Analysis at 1 years = the relationship between exposure in the first year of life and asthma onset between 1 and 6 years of age. Analysis at 2 years = the relationship between exposure in the first two years of life and asthma onset between 2 and 6 years of age. Analysis at 3 years = the relationship between exposure in the first three years of life and asthma onset between 3 and 6 years of age, etc. The reference group is unexposed with SPT-. SPT + =positive skin prick test; SPT- = negative skin prick test

A significant association between cumulative exposure during the first year of life and the risk of asthma onset the following year was observed, and a dose-response relation was indicated (Table 7). The results were similar when using number of hours only as the exposure variable (Additional file 8).

**Table 7:** Adjusted OR for asthma onset in the following year after cumulative exposure until specific ages

	Low-to intermediate exposure				High exposure			
	OR	(95% CI)			OR	(95% CI)		
1y (*n* = 60)	1.93	(1.01	–	3.68)	2.38	(1.13	–	5.00)
2y (*n* = 21)	2.37	(0.83	–	6.75)	1.07	(0.23	–	5.07)
3y (n = 17)	2.68	(0.71	–	10.13)	4.19	(1.23	–	14.30)

Analysis at 1 years = the relationship between exposure in the first year of life and asthma onset between 1 and 2 years of age. Analysis at 2 years = the relationship between exposure in the first two years of life and asthma onset between 2 and 3 years of age. Analysis at 3 years = the relationship between exposure in the first three years of life and asthma onset between 3 and 4 years of age

## Discussion

Our study showed that exposure to chlorinated swimming pool environments early in life was associated with an increased risk of pre-school asthma onset, especially so among the youngest children and those with an atopic predisposition. We analyzed the associations by using several different definitions of exposure before asthma onset, and all results pointed consistently in the same direction. A dose-response relationship between exposure and asthma onset between 1 and 2 years of age was indicated, although with limited statistical power. These findings may represent an opportunity to prevent allergic asthma [15].

The increased risk of asthma observed among the youngest children is in accordance with previous studies indicating that the youngest children were most susceptible [8, 11, 12]. In our study, the exposure levels of trichloramine were relatively low (mean 0.16 mg/m3), which may explain the lack of association with asthma onset at higher ages. More pronounced risks have been observed among sensitized children at higher trichloramine levels (0.3–0.5 mg/m3) [11]. The observations of trichloramine levels in swimming pool environments provided by the present study, fills a previously identified gap [7] in the literature on swimming pools and asthma. In our previous study [13] we found an association between swimming pool exposure and asthma among sensitized children. The present study complements these findings by observing that the youngest children had the highest risk of asthma and also specifies the trichloramine levels in the swimming pool environments. This additional information is important in order to relate our findings to observations by other scientists.

In the previous literature on respiratory disease and swimming pool exposure [7, 8, 10–14, 17–19, 26], some studies suggest an increased risk for asthma especially among children with atopy [8, 10–14] and in early life [8, 12, 26], findings in line with our results. The mechanism by which trichloramine may cause asthma is suggested to be through damaging the respiratory epithelium [8]. Children with sensitization to inhalant allergens might be vulnerable because of an existent inflammation in the lower airways, but trichloramine could also facilitate sensitization in previously non-atopic children by reducing the protective properties of the pulmonary epithelium [9–11, 27, 28]. There are also observations of increased risks of hay fever and eczema from swimming pool attendance, conditions with a prominent allergic component [17, 29, 30], strengthening the hypothesis of an interaction between disinfection by-products like trichloramine and inhalant allergens in causing asthma in children. However, other studies did not observe any increased risk of asthma, also when studying sensitized children [14, 17, 18]. Only one of these studies used measurements of trichloramine in combination with time spent in swimming pools as a marker of individual exposure, and merely 18% of those included in the study were characterized with regard to sensitization [14].

The present study is unique because it is based on a combination of a large and well-characterized population-based cohort with a very high participation rate, interview-based individual information on number of hours spent in indoor swimming pools, and objectively assessed trichloramine exposure. The nested case-control design made telephone interviewing feasible, and the participation rate was high in the interviews. The interviewed participants and the non-responders were very similar demographically, and were invited based on a well validated question on physician-diagnosed asthma [23]. We assessed exposure to trichloramine by multiplying the number of hours spent in indoor swimming pool with the mean trichloramine concentration in air in the swimming pool facility where the child went, and performed several sensitivity analyses that did not change the study interpretation. Data on sensitization status was available in a representative sample of more than 60% of the children. Furthermore, a major strength of our study design is that we were able to assess the cumulative exposure before the age of asthma onset.

There are however important limitations to our study, mainly regarding the retrospective estimation of exposure. Using a retrospectively reported item as part of the exposure assessment (number of hours) introduces a possible recall bias, i.e. parents to children with asthma may report more exposure to swimming pools. Also, the question on age at first asthma symptoms was asked retrospectively. However, the cases were initially based on those with physician-diagnosed asthma, and in all cases onset of asthma symptoms had occurred before the diagnosis. To the best of our knowledge, there is no awareness in the Swedish general population regarding swimming pools as a possible risk factor, why cases and controls should not differ regarding recall bias. Still, with our study design recall bias is not possible to rule out. There are also limitations regarding the exposure measurements. Air sampling was done during winter or spring in 2015, i.e. exposure measurements were in some cases collected many years from when the child attended the swimming pool facility, and in two premises measurements from 2005 and 2010, respectively, were used. There have been no major technical changes in the swimming pool facilities during the whole 16 year observation period, i.e. since the birth of the children. Still, the need to use current measurements to assess historical exposure is a limitation to our study. Earlier studies have shown a variability of personal exposure, based on personal or stationary sampling, to a chemical contaminant within or between individuals during a day or between different days at industrial premises [31], and one study reports that the air level of trichloramine in an indoor swimming pool increases during the day with the highest concentration in the evening [32]. We did not perform air sampling during late afternoon or in the evening as the children most often attended the swimming pool during day time. Our data (Table 3) indicates a variability of the personal exposure within or between the participating swimming pools. The variability can be caused by a random change in the exhaust ventilation rate, a difference in the emission of trichloramine from the pool water or other technical factors. On the other hand, children often visit the same pool multiple times and spend time in different parts of the facility, suggesting that the mean air level of trichloramine is a reasonable estimate of exposure. We found a correlation between free and bound chlorine in the water and the trichloramine concentration in air, but we did not find any correlation between the trichloramine level and the number of children attending the swimming pool. To evaluate the impact of different factors on the air concentration of trichloramine and thus a possible variability of the inhalation exposure over a longer time period in order to perform a retrospective exposure assessment demands a sophisticated, time consuming and expensive sampling strategy which was not within the scope of our study.

Thus, when performing the cumulative exposure assessment we had to assume that the data from our measurements give an acceptable estimate of the present exposure level as well as the exposure level during the past years when the children were also exposed. There is some support for this assumption as in the one swimming pool where there were two measurements made for the present study and older measurements of trichloramine available, the mean trichloramine concentrations were virtually identical. We have no data regarding the time the individuals spent in the pool water. Studies have however shown that the air concentration of trichloramine is similar at different spots within a pool area as well as close to the water surface [33].

The trichloramine concentrations in the included swimming pools were lower than in most previous studies where health risks have been identified. However, we have no measurements of trichloramine exposure specifically at baby swimming activities where higher water temperature may lead to higher concentrations of trichloramine. Therefore, the youngest children may have been exposed to higher trichloramine levels than our measurements indicate. Our analyses using only number of hours spent in indoor swimming pools, showed similar results as the analyses combining number of hours with objective measurements. However, the association between asthma onset at age 1 and cumulative exposure using objective measurements was somewhat stronger than in the analysis using numbers of hours only. The reasons for the similar results may be related to relatively low levels of trichloramine and small gradients of exposure.

Further, exposure in swimming pools may include other irritant gases [34]. In addition to trichloramine, monochloramine or dichloramine may also be present in the indoor air of swimming pools at approximately 10% of the total chloramine level (mono- di and trichloramine) [6]. We have not found any reports regarding human health effects following exposure to mono- and dichloramine.

In the analyses including sensitization status, the number of participants was relatively small, but the largely differing risk by sensitization status is still a very interesting finding in line with previous results on early life exposure and sensitization [8, 10–14]. We used SPT at age 7-8y as a marker of early atopic predisposition. A positive SPT was the strongest risk factor for asthma, as SPT alone yielded a markedly higher OR than exposure alone. However, the risk of asthma was about doubled for children with a combination of these two factors compared to children with a positive SPT but no exposure. The analyses were adjusted for a family history of asthma (both in siblings and parents), sex, geographical location (municipality) and maternal smoking (also during pregnancy). There was a difference in parental smoking between cases and controls and responders and non-responders. There was however no correlation between parental smoking and asthma at age 7-8y in the original cohort. As several other studies have found an association between maternal smoking and asthma, we chose to adjust the analyses for maternal smoking. Still, unmeasured confounding is possible. Moulds may be present in damp environments like swimming pool buildings, and has been linked to asthma in children [3]. We have no data regarding air concentration of mould, but when asking the technicians at the different premises they informed us that cleaning is taking place daily, thus minimizing the risk of moulds growing within the swimming pool area. However, moulds may be present in other parts of the premises, i.e. in the close vicinity of the filter system. The filters are placed in the cellar compartment, reducing the possibility that mould will contaminate the air in the pool area.

There might also be some factor related to asthma that makes parents more prone to take their children swimming, for example socioeconomic status or distance to the swimming pool, but it is unlikely that such a factor would mainly relate to sensitized children. Also, in this study area, a previous study showed no effect of distance to heavily trafficked roads on asthma among sensitized children [2], a risk factor that might be related to both place of living and socioeconomic status.

## Conclusions

This is the only study on childhood asthma and objectively measured trichloramine exposure at swimming pools that combines a longitudinal population-based design with individual information on exposure. Also after adjusting for possible confounding factors, we found a significantly increased risk of pre-school onset asthma from indoor swimming pool exposure, especially in children with atopic predisposition.

## Additional files

Additional file 1: Sampling and analysis of trichloramine. (DOCX 14 kb)

Additional file 2: Prevalence (%) of potential risk factors among responders and non-responders in the current case-control study. The data is based on the parental questionnaire to the entire cohort when the children were 7–8 years. (DOCX 13 kb)

Additional file 3: Unadjusted analyses: Pre-school onset vs controls (Unexposed as reference). (DOCX 15 kb)

Additional file 4: Adjusted OR for pre-school asthma in relation to cumulative exposure before asthma onset. Analyses based on Max instead of Mean exposure (Unexposed as reference). (DOCX 15 kb)

Additional file 5: Adjusted OR for pre-school asthma vs controls in relation to the cumulative exposure (hours*mean exposure) before asthma onset (Unexposed as reference) by excluding children attending swimming pools without objective measurements (“other swimming pools”). (DOCX 15 kb)

Additional file 6: Adjusted OR for pre-school asthma vs controls in relation to cumulative exposure before asthma onset. Analyses without municipality included in the model (Unexposed as reference). (DOCX 15 kb)

Additional file 7: Adjusted OR for pre-school asthma vs controls in relation to the exposure levels in swimming pools before asthma onset (Unexposed as reference). (DOCX 14 kb)

Additional file 8: Adjusted OR for asthma onset in the following year after cumulative exposure (number of hours) until specific ages. (DOCX 14 kb)

## Abbreviations

CI: confidence interval
EAACI: European Academy of Allergology and Clinical Immunology
ISAAC: The International Study of Asthma and Allergies in Childhood
NCl_3_: trichloramine
OLIN: Obstructive Lung Disease in Northern Sweden studies
OPC II: OLIN second pediatric cohort
OR: Odds ratio
SPT: Skin prick test
