# Supplementary material for: Early life swimming pool exposure and asthma onset in children – a case-control study
Source: Environ Health. 2018 Apr 11;17:34. doi: 10.1186/s12940-018-0383-0 (PMC5896097; doi:10.1186/s12940-018-0383-0)
Supplement: Supplementary file 1 — Sampling and analysis of trichloramine. (DOCX 14 kb) [file 12940_2018_383_MOESM1_ESM.docx]

Additional file 1

*Sampling and analysis of trichloramine*

Air sampling of trichloramine was performed using the method developed by Héry et al [25]. Trichloramine was collected using two glass fibre filters (Whatman Grade QMA, 37 mm diameter) impregnated with a solution of sodium carbonate (Na_2_CO_3_) and diarsenic trioxide (As_2_O_3_). The glass fibre filters were attached to a 37 mm filter plastic cassette connected to an air pump (Air Check 2000, SKC, USA) operated at an air flow of 2 l/min. The second filter was used to collect a possible overload of trichloramine on the first filter. Trichloramine is reduced to chloride ions (Cl^-^) on the impregnated filters. Following air sampling the filters were desorbed in 10 ml ultra-pure water (Direct-Q, UV, Millipore) in an ultrasonic bath during 10 minutes. The solution was filtered through a 13 mm syringe filter (IC Acrodisc^®^, Pall, USA). The chloride ions were analysed in a suppressed ion chromatography system, 788 IC filtration sample processor (Metrohm, Switzerland); 761 SD Compact IC (Metrohm, Switzerland); Conductivity detector ; Metrosep A Supp 5-100/4.0 anion column (Metrohm, Switzerland). The mobile phase was 10 mM NaOH with 25% acetone, and the suppressor was 5 mM H_2_SO_4_. The limit of detection (LOD) was 0.213 µg per sample corresponding to an air level of 0.001 mg/m^3^ with an air flow of 2 l/min and a sampling time of 90 minutes.
