# Supplementary material for: Early life swimming pool exposure and asthma onset in children – a case-control study
Source: Environ Health. 2018 Apr 11;17:34. doi: 10.1186/s12940-018-0383-0 (PMC5896097; doi:10.1186/s12940-018-0383-0)
Supplement: Supplementary file 2 — Prevalence (%) of potential risk factors among responders and non-responders in the current case-control study. The data is based on the parental questionnaire to the entire cohort when the children were 7–8 years. (DOCX 13 kb) [file 12940_2018_383_MOESM2_ESM.docx]

Additional file 2

| **Prevalence (%) of potential risk factors among responders and non-responders in the current case-control study. The data is based on the parental questionnaire to the entire cohort when the children were 7-8 years.** | | | |
| --- | --- | --- | --- |
|  |  |  |  |
|  | Cases (n=337) | Controls (n=633) | p |
| Paternal smoking | 12.6 | 11.8 | 0.75 |
| Maternal smoking | 14.8 | 11.8 | 0.55 |
| Family history of asthma | 40.4% | 17.1% | <0.001 |
| Family history of allergy | 52.8% | 31.1% | <0.001 |
| Urban living | 70.2% | 64.6% | 0.10 |
| Number of siblings (mean) | 1.60 | 1.57 | 0.67 |
|  |  |  |  |
