# Supplementary material for: Early life swimming pool exposure and asthma onset in children – a case-control study
Source: Environ Health. 2018 Apr 11;17:34. doi: 10.1186/s12940-018-0383-0 (PMC5896097; doi:10.1186/s12940-018-0383-0)
Supplement: Supplementary file 3 — Unadjusted analyses: Pre-school onset vs controls (Unexposed as reference). (DOCX 15 kb) [file 12940_2018_383_MOESM3_ESM.docx]

Additional file 3

| **Unadjusted OR for pre-school asthma vs controls in relation to cumulative exposure before asthma onset (Unexposed as reference)** | | | | | | | | | | | | | | | |
| --- | --- | --- | --- | --- | --- | --- | --- | --- | --- | --- | --- | --- | --- | --- | --- |
|  | Low-to intermediate exposure | | | |  | High exposure | | | | | |  | Any exposure | | |
| Age | OR | | (95% CI) | |  | OR | (95% CI) | | | | |  | OR | (95% CI) | |
| 1y (n=153) | 1.53 | | (1.02 | 2.31) |  | 1.74 | (1.05 | | | 2.88) | |  | 1.60 | (1.12 | 2.29) |
| 2y (n=93) | 1.62 | | (1.00 | 2.61) |  | 1.26 | (0.67 | | | 2.37) | |  | 1.49 | (0.96 | 2.31) |
| 3y (n=72) | 1.58 | | (0.92 | 2.72) |  | 1.20 | (0.60 | | | 2.41) | |  | 1.45 | (0.87 | 2.40) |
| 4y (n=55) | 1.08 | | (0.58 | 2.02) |  | 0.93 | (0.43 | | | 2.02) | |  | 1.03 | (0.57 | 1.85) |
| 5y (n=39) | 0.85 | | (0.39 | 1.82) |  | 0.81 | (0.33 | | | 2.00) | |  | 0.83 | (0.41 | 1.71) |
| 6y (n=26) | 0.66 | | (0.23 | 1.93) |  | 0.98 | (0.32 | | | 2.99) | |  | 0.77 | (0.28 | 2.09) |
|  |  |  |  |  |  |  | |  |  | |  |  |  | |  |

*Exposure=Hours*Mean Cumulative Exposure level*

Footnote: Analysis at 1 years=the relationship between exposure in the first year of life and asthma onset between 1 and 6 years of age (pre-school asthma). Analysis at 2 years=the relationship between exposure in the first two years of life and asthma onset between 2 and 6 years of age. Analysis at 3 years=the relationship between exposure in the first three years of life and asthma onset between 3 and 6 years of age, etc.
