# Supplementary material for: Early life swimming pool exposure and asthma onset in children – a case-control study
Source: Environ Health. 2018 Apr 11;17:34. doi: 10.1186/s12940-018-0383-0 (PMC5896097; doi:10.1186/s12940-018-0383-0)
Supplement: Supplementary file 4 — Adjusted OR for pre-school asthma in relation to cumulative exposure before asthma onset. Analyses based on Max instead of Mean exposure (Unexposed as reference). (DOCX 15 kb) [file 12940_2018_383_MOESM4_ESM.docx]

Additional file 4

| **Adjusted OR for pre-school asthma in relation to cumulative exposure before asthma onset. Analyses based on Max instead of Mean exposure (Unexposed as reference)** | | | | | | | | | | | | | |
| --- | --- | --- | --- | --- | --- | --- | --- | --- | --- | --- | --- | --- | --- |
|  | Low-to intermediate exposure | | |  | High exposure | | | | |  | Any exposure | | |
|  | OR | (95% CI) | |  | OR | (95% CI) | | | |  | OR | (95% CI) | |
| 1y (n=153) | 1.85 | (1.17 | 2.91) |  | 2.12 | (1.25 | 3.59) | | |  | 1.95 | (1.33 | 2.86) |
| 2y (n=93) | 1.89 | (1.11 | 3.21) |  | 1.69 | (0.88 | 3.25) | | |  | 1.82 | (1.14 | 2.90) |
| 3y (n=72) | 1.79 | (1.00 | 3.22) |  | 1.48 | (0.73 | 3.02) | | |  | 1.68 | (0.98 | 2.86) |
| 4y (n=55) | 1.27 | (0.66 | 2.45) |  | 1.04 | (0.46 | 2.36) | | |  | 1.19 | (0.65 | 2.20) |
| 5y (n=39) | 1.12 | (0.49 | 2.52) |  | 1.14 | (0.45 | 2.90) | | |  | 1.13 | (0.53 | 2.39) |
| 6y (n=26) | 0.87 | (0.29 | 2.59) |  | 1.10 | (0.33 | 3.63) | | |  | 0.94 | (0.34 | 2.65) |
|  |  |  |  |  |  |  |  | | |  |  |  |  |
| *Exposure=Hours*Max Cumulative Exposure level* | | | | | | |  |  |  |  |  | | |

Footnote: Analysis at 1 years=the relationship between exposure in the first year of life and asthma onset between 1 and 6 years of age. Analysis at 2 years=the relationship between exposure in the first two years of life and asthma onset between 2 and 6 years of age. Analysis at 3 years=the relationship between exposure in the first three years of life and asthma onset between 3 and 6 years of age, etc.
