# Supplementary material for: Early life swimming pool exposure and asthma onset in children – a case-control study
Source: Environ Health. 2018 Apr 11;17:34. doi: 10.1186/s12940-018-0383-0 (PMC5896097; doi:10.1186/s12940-018-0383-0)
Supplement: Supplementary file 5 — Adjusted OR for pre-school asthma vs controls in relation to the cumulative exposure (hours*mean exposure) before asthma onset (Unexposed as reference) by excluding children attending swimming pools without objective measurements (“other swimming pools”). (DOCX 15 kb) [file 12940_2018_383_MOESM5_ESM.docx]

Additional file 5

| **Adjusted OR for pre-school asthma vs controls in relation to the cumulative exposure (hours*mean exposure) before asthma onset (Unexposed as reference) by excluding children attending swimming pools without objective measurements (“other swimming pools”)** | | | | | | | | | | | |
| --- | --- | --- | --- | --- | --- | --- | --- | --- | --- | --- | --- |
|  | Low-to intermediate exposure | | |  | High exposure | | |  | Any exposure | | |
| Age | OR | (95% CI) | |  | OR | (95% CI) | |  | OR | (95% CI) | |
| 1y (n=136) | 1.6 | 1.03 | 2.6 |  | 1.3 | 0.6 | 1.8 |  | 1.5 | 1.01 | 2.3 |
| 2y (n=79) | 1.7 | 0.99 | 3.0 |  | 0.8 | 0.3 | 1.7 |  | 1.3 | 0.8 | 2.2 |
| 3y (n=60) | 1.5 | 0.8 | 2.7 |  | 0.9 | 0.4 | 2.1 |  | 1.3 | 0.7 | 2.2 |
| 4y (n=46) | 1.0 | 0.5 | 1.9 |  | 0.8 | 0.3 | 2.0 |  | 0.9 | 0.5 | 1.8 |
| 5y (n=32) | 0.9 | 0.4 | 2.2 |  | 0.7 | 0.2 | 2.0 |  | 0.9 | 0.4 | 1.9 |
| 6y (n=23) | 0.8 | 0.3 | 2.4 |  | 0.8 | 0.2 | 3.0 |  | 0.8 | 0.3 | 2.3 |
|  |  |  |  |  |  |  |  |  |  |  |  |

*Exposure=Hours*Mean Cumulative Exposure level*

Footnote: Analysis at 1 years=the relationship between exposure in the first year of life and asthma onset between 1 and 6 years of age. Analysis at 2 years=the relationship between exposure in the first two years of life and asthma onset between 2 and 6 years of age. Analysis at 3 years=the relationship between exposure in the first three years of life and asthma onset between 3 and 6 years of age, etc.
