# Supplementary material for: Early life swimming pool exposure and asthma onset in children – a case-control study
Source: Environ Health. 2018 Apr 11;17:34. doi: 10.1186/s12940-018-0383-0 (PMC5896097; doi:10.1186/s12940-018-0383-0)
Supplement: Supplementary file 6 — Adjusted OR for pre-school asthma vs controls in relation to cumulative exposure before asthma onset. Analyses without municipality included in the model (Unexposed as reference). (DOCX 15 kb) [file 12940_2018_383_MOESM6_ESM.docx]

Additional file 6

| **Adjusted OR for pre-school asthma vs controls in relation to cumulative exposure before asthma onset. Analyses without municipality included in the model (Unexposed as reference)** | | | | | | | | | | | | | | |
| --- | --- | --- | --- | --- | --- | --- | --- | --- | --- | --- | --- | --- | --- | --- |
|  | Low-to intermediate exposure | | |  | High exposure | | | | | |  | Any exposure | | |
| Age | OR | (95% CI) | |  | OR | (95% CI) | | | | |  | OR | (95% CI) | |
| 1y (n=153) | 1.97 | (1.28 | 3.05) |  | 1.94 | (1.14 | 3.32) | | | |  | 1.96 | (1.34 | 2.87) |
| 2y (n=93) | 2.04 | (1.22 | 3.39) |  | 1.43 | (0.74 | 2.78) | | | |  | 1.82 | (1.14 | 2.89) |
| 3y (n=72) | 1.93 | (1.08 | 3.42) |  | 1.27 | (0.61 | 2.64) | | | |  | 1.68 | (0.99 | 2.87) |
| 4y (n=55) | 1.28 | (0.66 | 2.45) |  | 1.01 | (0.45 | 2.26) | | | |  | 1.18 | (0.64 | 2.18) |
| 5y (n=39) | 1.21 | (0.54 | 2.70) |  | 0.97 | (0.38 | 2.50) | | | |  | 1.12 | (0.53 | 2.38) |
| 6y (n=26) | 0.82 | (0.27 | 2.47) |  | 1.14 | (0.36 | 3.61) | | | |  | 0.93 | (0.33 | 2.61) |
|  |  |  |  |  |  |  |  | | | |  |  |  |  |
| *Exposure=Hours*Mean Cumulative Exposure level* | | | | | | | |  |  |  |  |  | | |

Footnote: Analysis at 1 years=the relationship between exposure in the first year of life and asthma onset between 1 and 6 years of age. Analysis at 2 years=the relationship between exposure in the first two years of life and asthma onset between 2 and 6 years of age. Analysis at 3 years=the relationship between exposure in the first three years of life and asthma onset between 3 and 6 years of age, etc.
