# Supplementary material for: Early life swimming pool exposure and asthma onset in children – a case-control study
Source: Environ Health. 2018 Apr 11;17:34. doi: 10.1186/s12940-018-0383-0 (PMC5896097; doi:10.1186/s12940-018-0383-0)
Supplement: Supplementary file 7 — Adjusted OR for pre-school asthma vs controls in relation to the exposure levels in swimming pools before asthma onset (Unexposed as reference). (DOCX 14 kb) [file 12940_2018_383_MOESM7_ESM.docx]

Additional file 7

| **Adjusted OR for pre-school asthma vs controls in relation to the exposure levels in swimming pools before asthma onset (Unexposed as reference)** | | | | | | | | | | | |
| --- | --- | --- | --- | --- | --- | --- | --- | --- | --- | --- | --- |
|  | Low-to intermediate exposure | | |  | High exposure | | |  | Any exposure | | |
| Age | OR | (95% CI) | |  | OR | (95% CI) | |  | OR | (95% CI) | |
| 1y (n=153) | 1.92 | (1.24 | 3.00) |  | 2.00 | (1.14 | 3.52) |  | 1.95 | (1.33 | 2.86) |
| 2y (n=93) | 2.03 | (1.22 | 3.39) |  | 1.40 | (0.69 | 2.84) |  | 1.82 | (1.14 | 2.90) |
| 3y (n=72) | 1.77 | (0.998 | 3.12) |  | 1.46 | (0.68 | 3.17) |  | 1.68 | (0.98 | 2.86) |
|  | |  |  |  |  | | |  |  | |  |

*Exposure= Mean Cumulative Exposure level*

Footnote: Analysis at 1 years=the relationship between exposure in the first year of life and asthma onset between 1 and 6 years of age. Analysis at 2 years=the relationship between exposure in the first two years of life and asthma onset between 2 and 6 years of age. Analysis at 3 years=the relationship between exposure in the first three years of life and asthma onset between 3 and 6 years of age, etc.
