# Supplementary material for: Early life swimming pool exposure and asthma onset in children – a case-control study
Source: Environ Health. 2018 Apr 11;17:34. doi: 10.1186/s12940-018-0383-0 (PMC5896097; doi:10.1186/s12940-018-0383-0)
Supplement: Supplementary file 8 — Adjusted OR for asthma onset in the following year after cumulative exposure (number of hours) until specific ages. (DOCX 14 kb) [file 12940_2018_383_MOESM8_ESM.docx]

Additional file 8

| **Adjusted OR for asthma onset in the following year after cumulative exposure (number of hours) until specific ages.** | | | | | | | | | |
| --- | --- | --- | --- | --- | --- | --- | --- | --- | --- |
|  | Low-to-intermediate exposure | | | |  | High exposure | | | |
|  | OR | (95% CI) | | |  | OR | (95% CI) | | |
| 1y (n=60) | 1.78 | (0.84 | - | 3.75) |  | 2.33 | (1.24 | - | 4.37) |
| 2y (n=21) | 3.19 | (1.08 | - | 9.39) |  | 1.00 | (0.27 | - | 3.71) |
| 3y (n=17) | 4.12 | (1.09 | - | 15.57) |  | 2.98 | (0.89 | - | 9.93) |

*Exposure=Cumulative number of hours*

Footnote: Analysis at 1 years=the relationship between exposure in the first year of life and asthma onset between 1 and 2 years of age. Analysis at 2 years=the relationship between exposure in the first two years of life and asthma onset between 2 and 3 years of age. Analysis at 3 years=the relationship between exposure in the first three years of life and asthma onset between 3 and 4 years of age.
